# Supplementary material for: Analysis of PPARγ Signaling Activity in Psoriasis
Source: Int J Mol Sci. 2021 Aug 10;22(16):8603. doi: 10.3390/ijms22168603 (PMC8395241; doi:10.3390/ijms22168603)
Supplement: Supplementary file 1 [file ijms-22-08603-s001.zip › Supplemental materials_Analysis of PPARg signaling activity in psoriasis/Pathway models/Models images and html files/Model of PPARG signaling in psoriasis/4474.html]

FLT1


# Protein FLT1

|  |  |
| --- | --- |
| URN | urn:agi-llid:2321 |
| Total Entities | 0 |
| Connectivity | 4262 |
| Name | FLT1 |
| Description | fms related tyrosine kinase 1 |
| Notes | This gene encodes a member of the vascular endothelial growth factor receptor (VEGFR) family. VEGFR family members are receptor tyrosine kinases (RTKs) which contain an extracellular ligand-binding region with seven immunoglobulin (Ig)-like domains, a transmembrane segment, and a tyrosine kinase (TK) domain within the cytoplasmic domain. This protein binds to VEGFR-A, VEGFR-B and placental growth factor and plays an important role in angiogenesis and vasculogenesis. Expression of this receptor is found in vascular endothelial cells, placental trophoblast cells and peripheral blood monocytes. Multiple transcript variants encoding different isoforms have been found for this gene. Isoforms include a full-length transmembrane receptor isoform and shortened, soluble isoforms. The soluble isoforms are associated with the onset of pre-eclampsia.[provided by RefSeq, May 2009] |
| Primary Cell Localization | Plasma membrane |
| Class | Receptor |

---

|  |  |
| --- | --- |
| Pathway | VEGFA/NOTCH1/WNT Cross-talk in Blood Vessel Sprouting and Branching in Cancer |
|  | Proteins Involved in Arterial Hypertension |
|  | Proteins with Altered Expression in Melanoma |
|  | Melanoma |
|  | Proteins Involved in Melanoma |
|  | Endothelial Cell Dysfunction in Progressive Diabetic Nephropathy |
|  | Proliferative Diabetic Retinopathy |
|  | Cetuximab Resistance in Colorectal Cancer |
|  | Proteins Involved in Hepatocellular Carcinoma |
|  | Hepatocellular Carcinoma |
|  | Growth Factor Signaling in Hepatocellular Carcinoma |
|  | Proteins Involved in Pancreatic Cancer |
|  | Pancreatic Ductal Carcinoma |
|  | Growth Factor Signaling in Pancreatic Neoplasms |
|  | Proteins Involved in Astrocytoma |
|  | Proteins Involved in Breast Cancer Related to ERBB2/VEGFR/Akt Signaling Pathway |
|  | ERBB/VEGFR/Akt Signaling in Breast Cancer |
|  | ESR1/ERBB Positive Luminal Breast Cancer |
|  | Proteins with Altered Expression in Breast Cancer |
|  | Breast Cancer |
|  | Endometrial Cancer |
|  | Proteins Involved in Endometrial Cancer |
|  | Proteins with Altered Expression in Endometrial Cancer |
|  | Proteins Involved in Endometriosis |
|  | Angiogenesis in Endometriosis |
|  | Proteins Involved in Spontaneous Abortion |
|  | Proteins Involved in High-Grade Serous Ovarian Carcinoma |
|  | High-Grade Serous Ovarian Carcinoma |
|  | Ovarian Cancer |
|  | Proteins with Altered Expression in Ovarian Cancer |
|  | Netrin-1 Signaling in Age-Related Macular Degeneration |
|  | Proteins Involved in Age-Related Macular Degeneration |
|  | HIF1A in Pathologic Ocular Neovascularization |
|  | VEGFA in Neovascular Glaucoma |
|  | VEGFA Low Expression Level in Chronic Bronchitis |
|  | CSF2 -> NF-kB Expression Targets |
|  | EGF -> CTNN Expression Targets |
|  | EGF -> AP-1/ATF Expression Targets |
|  | EGF -> CREB/CREBBP/ELK/SRF/MYC Expression Targets |
|  | EGF -> MEF/MYOD/NFATC Expression Targets |
|  | F2 -> AP-1/CREB/ELK/SRF/SP1 Expression Targets |
|  | F2 -> STAT1/NF-kB Expression Targets |
|  | FGF2 -> STAT Expression Targets |
|  | FGF2 -> AP-1/CREB/CREBBP/ELK/SRF/MYC Expression Targets |
|  | VIP Expression Targets |
|  | HGF -> STAT Expression Targets |
|  | HGF -> AP-1/CREB/ELK/SRF/MYC Expression Targets |
|  | HIF1A in Vasculogenic Mimicry of Cancer |
|  | HIF1A as Master Regulator of Angiogenesis in Cancer |
|  | INHBA/ACVR2/BMPR Expression Targets |
|  | Insulin -> STAT Expression Targets |
|  | Insulin -> CEBPA/CTNNB/FOXA/FOXO Expression Targets |
|  | Insulin -> MEF/MYOD Expression Targets |
|  | Insulin -> ELK/SRF/HIF1A/MYC/SREBF Expression Targets |
|  | NGF -> AP-1/TP53/MYC Expression Targets |
|  | AGT -> TP53 Expression Targets |
|  | EDN1 Expression Targets |
|  | AGT -> CREB Expression Targets |
|  | FIGF -> AP-1 Expression Target |
|  | FIGF -> NCOR2 Expression Target |
|  | PGF -> AP-1/CREB/CREBBP/MYC Expression Targets |
|  | Elevated Receptors -> Expression Targets in Placenta |
|  | VEGF Signaling |
|  | TGFB1-TGFBR2 Expression Targets |
|  | TGFB1-TGFBR1/AP-1 Expression Targets |
|  | TGFB1-ACVRL1 Expression Targets |
|  | IL1B Expression Targets |
|  | VEGFA -> ATF/CREB/ELK-SRF Expression Targets |
|  | VEGFC -> CTNNB Expression Target |
|  | VEGFC -> ATF Expression Target |
|  | VEGFA -> FOXO3A Expression Targets |
|  | VEGFA -> AP-1/CREBBP/MYC Expression Targets |
|  | VEGFA -> CTNNB/CTNND Expression Targets |
|  | VEGFA -> NCOR2 Expression Target |
|  | VEGFR -> CTNNB Signaling |
|  | VEGFR -> AP-1/CREB/MYC Signaling |
|  | VEGFR -> FOXO3A Signaling |
|  | VEGFR -> ATF/CREB/ELK-SRF Signaling |
|  | pprarg neg, uknown targets, ps-positive |
|  | prarg neg,ukn expres targets, ps-positive |
|  | PPARG negative targets |
|  | pparg expr target\_regulators |
|  | Neighbors of disease exacerbation |
|  | Neighbors of tumor progression |
|  | Neighbors of cancer progression |
|  | Neighbors of inflammatory status |
|  | Neighbors of overall survival |
|  | Neighbors of disease severity |
|  | Neighbors of tumor size |
|  | Neighbors of mortality |
|  | Neighbors of survival rate |
|  | Neighbors of infarct size |
|  | Neighbors of survival time |
|  | Neighbors of functional recovery |
|  | Neighbors of insulin sensitivity |
|  | Neighbors of cancer survival |
|  | Neighbors of graft survival |
|  | Neighbors of therapeutic efficacy |
|  | Neighbors of lethality |
|  | Neighbors of tumor regression |
|  | Neighbors of disease-free survival |
|  | Neighbors of macrophage count |
|  | Neighbors of treatment outcome |
|  | Neighbors of bone mass |
|  | Neighbors of body weight |
|  | Neighbors of body weight gain |
|  | Neighbors of cardiovascular risk |
|  | Neighbors of microvessel density |
|  | Neighbors of tumor recurrence |
|  | Neighbors of glucose tolerance |
|  | Neighbors of morbidity |
|  | Neighbors of fibrosis progression |
|  | Neighbors of mortality rate |
|  | Neighbors of fat mass |
|  | Neighbors of adverse outcome |
|  | Neighbors of lesion area |
|  | Neighbors of cell density |
|  | Neighbors of progression-free survival |
|  | Neighbors of apoptosis rate |
|  | Neighbors of early mortality |
|  | Neighbors of blood pressure |
|  | Neighbors of leukocyte count |
|  | Neighbors of vascular reactivity |
|  | Neighbors of vascular leakage |
|  | Neighbors of body mass index |
|  | Neighbors of vascular density |
|  | Neighbors of tumor aggressiveness |
|  | Neighbors of systolic blood pressure |
|  | Neighbors of tumor resistance |
|  | Neighbors of renal hypertrophy |
|  | Neighbors of vascular tone |
|  | Neighbors of proliferation index |
|  | Neighbors of cell permeability |
|  | Neighbors of pregnancy outcome |
|  | Neighbors of fertility |
|  | Neighbors of fractional shortening |
|  | Neighbors of disease exacerbation |
|  | Neighbors of tumor progression |
|  | Neighbors of cancer progression |
|  | Neighbors of inflammatory status |
|  | Neighbors of overall survival |
|  | Neighbors of disease severity |
|  | Neighbors of tumor size |
|  | Neighbors of mortality |
|  | Neighbors of survival rate |
|  | Neighbors of infarct size |
|  | Neighbors of survival time |
|  | Neighbors of functional recovery |
|  | Neighbors of insulin sensitivity |
|  | Neighbors of cancer survival |
|  | Neighbors of graft survival |
|  | Neighbors of therapeutic efficacy |
|  | Neighbors of lethality |
|  | Neighbors of tumor regression |
|  | Neighbors of disease-free survival |
|  | Neighbors of macrophage count |
|  | Neighbors of treatment outcome |
|  | Neighbors of bone mass |
|  | Neighbors of body weight |
|  | Neighbors of body weight gain |
|  | Neighbors of cardiovascular risk |
|  | Neighbors of microvessel density |
|  | Neighbors of tumor recurrence |
|  | Neighbors of glucose tolerance |
|  | Neighbors of morbidity |
|  | Neighbors of fibrosis progression |
|  | Neighbors of mortality rate |
|  | Neighbors of fat mass |
|  | Neighbors of adverse outcome |
|  | Neighbors of lesion area |
|  | Neighbors of cell density |
|  | Neighbors of progression-free survival |
|  | Neighbors of apoptosis rate |
|  | Neighbors of early mortality |
|  | Neighbors of blood pressure |
|  | Neighbors of leukocyte count |
|  | Neighbors of vascular reactivity |
|  | Neighbors of vascular leakage |
|  | Neighbors of body mass index |
|  | Neighbors of vascular density |
|  | Neighbors of tumor aggressiveness |
|  | Neighbors of systolic blood pressure |
|  | Neighbors of tumor resistance |
|  | Neighbors of renal hypertrophy |
|  | Neighbors of vascular tone |
|  | Neighbors of proliferation index |
|  | Neighbors of cell permeability |
|  | Neighbors of pregnancy outcome |
|  | Neighbors of fertility |
|  | Neighbors of fractional shortening |
|  | Neighbors of cell development |
|  | Neighbors of immune response |
|  | Neighbors of cell population |
|  | Neighbors of T-cell development |
|  | Neighbors of inflammatory response |
|  | Neighbors of wound healing |
|  | Neighbors of angiogenesis |
|  | Neighbors of cell function |
|  | Neighbors of macrophage activation |
|  | Neighbors of cell formation |
|  | Neighbors of osteoclast development |
|  | Neighbors of epithelial to mesenchymal transition |
|  | Neighbors of immunity |
|  | Neighbors of cell proliferative response |
|  | Neighbors of chemotaxis |
|  | Neighbors of dendritic cell differentiation |
|  | Neighbors of cell survival |
|  | Neighbors of osteoclast differentiation |
|  | Neighbors of cell phenotype |
|  | Neighbors of T-cell proliferation |
|  | Neighbors of tissue remodeling |
|  | Neighbors of cell adhesion |
|  | Neighbors of T-cell activation |
|  | Neighbors of endothelial cell proliferation |
|  | Neighbors of fibrogenesis |
|  | Neighbors of tumor growth |
|  | Neighbors of dendritic cell development |
|  | Neighbors of tissue repair |
|  | Neighbors of cell count |
|  | Neighbors of cell infiltration |
|  | Neighbors of cell interaction |
|  | Neighbors of SMC proliferation |
|  | Neighbors of lumen formation |
|  | Neighbors of endothelial cell migration |
|  | Neighbors of adipogenesis |
|  | Neighbors of lipid storage |
|  | Neighbors of cell damage |
|  | Neighbors of pregnancy |
|  | Neighbors of monocyte differentiation |
|  | Neighbors of aging |
|  | Neighbors of cell motility |
|  | Neighbors of monocyte migration |
|  | Neighbors of macrophage migration |
|  | Neighbors of microglial activation |
|  | Neighbors of regeneration |
|  | Neighbors of cell migration |
|  | Neighbors of cancer growth |
|  | Neighbors of stem cell proliferation |
|  | Neighbors of smooth muscle cell migration |
|  | Neighbors of hepatic regeneration |
|  | Neighbors of phagocytosis |
|  | Neighbors of immune system activation |
|  | Neighbors of leukocyte migration |
|  | Neighbors of leukocyte recruitment |
|  | Neighbors of osteoclast formation |
|  | Neighbors of monocyte recruitment |
|  | Neighbors of cell invasion |
|  | Neighbors of heart function |
|  | Neighbors of hemopoiesis |
|  | Neighbors of bone remodeling |
|  | Neighbors of ossification |
|  | Neighbors of immune system function |
|  | Neighbors of lung development |
|  | Neighbors of autophagy |
|  | Neighbors of monocyte adhesion |
|  | Neighbors of senescence |
|  | Neighbors of hepatocyte proliferation |
|  | Neighbors of cell transdifferentiation |
|  | Neighbors of neuroprotection |
|  | Neighbors of stem cell differentiation |
|  | Neighbors of endothelial cell function |
|  | Neighbors of neuronal death |
|  | Neighbors of cell growth |
|  | Neighbors of ROS generation |
|  | Neighbors of epithelial cell proliferation |
|  | Neighbors of first trimester pregnancy |
|  | Neighbors of cancer cell growth |
|  | Neighbors of sensitization |
|  | Neighbors of macrophage function |
|  | Neighbors of cellular senescence |
|  | Neighbors of colony formation |
|  | Neighbors of cell differentiation |
|  | Neighbors of stem cell migration |
|  | Neighbors of life span |
|  | Neighbors of cell homeostasis |
|  | Neighbors of inflammatory disease |
|  | Neighbors of rheumatoid arthritis |
|  | Neighbors of atherosclerosis |
|  | Neighbors of fibrosis |
|  | Neighbors of colitis |
|  | Neighbors of arthritis |
|  | Neighbors of inflammatory bowel disease |
|  | Neighbors of injury |
|  | Neighbors of atherogenesis |
|  | Neighbors of vascular remodeling |
|  | Neighbors of metastasis |
|  | Neighbors of carcinogenesis |
|  | Neighbors of inflammation |
|  | Neighbors of tumor microenvironment |
|  | Neighbors of pathological angiogenesis |
|  | Neighbors of obesity |
|  | Neighbors of vascular disease |
|  | Neighbors of diabetes mellitus |
|  | Neighbors of pneumonia |
|  | Neighbors of hyperplasia |
|  | Neighbors of osteoarthritis |
|  | Neighbors of liver fibrosis |
|  | Neighbors of myocardial infarction |
|  | Neighbors of leukocyte infiltration |
|  | Neighbors of airway inflammation |
|  | Neighbors of infection |
|  | Neighbors of vascular injury |
|  | Neighbors of renal injury |
|  | Neighbors of preeclampsia |
|  | Neighbors of macrophage infiltration |
|  | Neighbors of liver injury |
|  | Neighbors of sepsis |
|  | Neighbors of death |
|  | Neighbors of type 1 diabetes |
|  | Neighbors of ischemic stroke |
|  | Neighbors of heart disease |
|  | Neighbors of diabetic nephropathy |
|  | Neighbors of lung injury |
|  | Neighbors of immunopathology |
|  | Neighbors of systemic sclerosis |
|  | Neighbors of hepatocellular carcinoma |
|  | Neighbors of acute lung injury |
|  | Neighbors of gastric cancer |
|  | Neighbors of atherosclerotic plaque |
|  | Neighbors of experimental autoimmune encephalomyelitis |
|  | Neighbors of colorectal cancer |
|  | Neighbors of cardiac remodeling |
|  | Neighbors of breast cancer |
|  | Neighbors of ischemia |
|  | Neighbors of melanoma |
|  | Neighbors of collagen-induced arthritis |
|  | Neighbors of neoplasm invasion |
|  | Neighbors of kidney disease |
|  | Neighbors of heart failure |
|  | Neighbors of lung disease |
|  | Neighbors of cardiovascular disease |
|  | Neighbors of stroke |
|  | Neighbors of cancer |
|  | Neighbors of cerebral ischemia |
|  | Neighbors of neoplasm |
|  | Neighbors of endometriosis |
|  | Neighbors of neointima |
|  | Neighbors of vasculitis |
|  | Neighbors of diet-induced obesity |
|  | Neighbors of myocardial fibrosis |
|  | Neighbors of coronary artery disease |
|  | Neighbors of rupture |
|  | Neighbors of endothelial cell dysfunction |
|  | Neighbors of prostate cancer |
|  | Neighbors of hypertrophy |
|  | Neighbors of osteolysis |
|  | Neighbors of ovarian cancer |
|  | Neighbors of pancreatic cancer |
|  | Neighbors of hypertension |
|  | Neighbors of proteinuria |
|  | Neighbors of epithelial cell |
|  | Neighbors of vascular smooth muscle cell |
|  | Neighbors of umbilical vein endothelial cell |
|  | Neighbors of monocyte |
|  | Neighbors of endometrial stromal cell |
|  | Neighbors of alveolar macrophage |
|  | Neighbors of macrophage |
|  | Neighbors of adipocyte |
|  | Neighbors of fibroblast |
|  | Neighbors of PBMC |
|  | Neighbors of stromal cell |
|  | Neighbors of trophoblast |
|  | Neighbors of mononuclear cell |
|  | Neighbors of endothelial cell |
|  | Neighbors of dendritic cell |
|  | Neighbors of kidney tubule cell |
|  | Neighbors of inflammatory cell |
|  | Neighbors of bone marrow macrophage |
|  | Neighbors of neutrophil |
|  | Neighbors of smooth muscle myocyte |
|  | Neighbors of activated T-cell |
|  | Neighbors of mononuclear phagocyte |
|  | Neighbors of capillary endothelial cell |
|  | Neighbors of immunocompetent cell |
|  | Neighbors of cardiac myocyte |
|  | Neighbors of myeloid cell |
|  | Neighbors of mesenchymal stroma cell |
|  | Neighbors of corneal epithelial cell |
|  | Neighbors of cytotrophoblast |
|  | Neighbors of stem cell |
|  | Neighbors of B-cell |
|  | Neighbors of granulosa cell |
|  | Neighbors of leukocyte |
|  | Neighbors of insulin-secreting cell |
|  | Neighbors of hematopoietic cell |
|  | Neighbors of podocyte |
|  | Neighbors of pericyte |
|  | Neighbors of kidney cell |
|  | Neighbors of syncytiotrophoblast |
|  | Neighbors of lymphocyte |
|  | Neighbors of retina cell |
|  | Neighbors of lung |
|  | Neighbors of liver |
|  | Neighbors of kidney |
|  | Neighbors of lymph node |
|  | Neighbors of blood vessel |
|  | Neighbors of heart |
|  | Neighbors of microvessel |
|  | Neighbors of placenta |
|  | Neighbors of carotid artery |
|  | Neighbors of uterus |
|  | Neighbors of retina |
|  | Neighbors of omentum |
|  | Neighbors of arteriole |
|  | Neighbors of villus |
|  | Neighbors of eye |
|  | Neighbors of hippocampus |
|  | Neighbors of cerebral cortex |
|  | Neighbors of bladder |
|  | Neighbors of Fallopian tube |
|  | Neighbors of NF-kB family |
|  | Neighbors of oxidized LDL |
|  | Neighbors of JNK |
|  | Neighbors of PPAR |
|  | Neighbors of Jun/Fos |
|  | Neighbors of PKC |
|  | Neighbors of PI3K |
|  | Neighbors of mitogen-activated protein kinase |
|  | Neighbors of TLR |
|  | Neighbors of cytokine |
|  | Neighbors of JAK |
|  | Neighbors of NF-AT family |
|  | Neighbors of beta adrenoceptor |
|  | Neighbors of chorionic gonadotropin |
|  | Neighbors of adipokine |
|  | Neighbors of NAD(P)H oxidase |
|  | Neighbors of IgG |
|  | Neighbors of SMAD subfamily |
|  | Neighbors of inflammatory cytokine |
|  | Neighbors of estrogen receptor |
|  | Neighbors of PDGF-BB |
|  | Neighbors of HIF-1 |
|  | Neighbors of GPCR |
|  | Neighbors of IL12 |
|  | Neighbors of Notch |
|  | Neighbors of heat shock protein 90 |
|  | Neighbors of fibrinogen |
|  | Neighbors of interleukin-35 |
|  | Neighbors of Ang II receptor |
|  | Neighbors of proteasome endopeptidase complex |
|  | Neighbors of alpha2 adrenoceptor |
|  | Neighbors of vitronectin receptor (integrin) |
|  | Neighbors of WNT |
|  | Neighbors of gonadotropins |
|  | Neighbors of PP2B |
|  | Neighbors of AHR subfamily |
|  | Neighbors of endostatins |
|  | Neighbors of relaxin |
|  | comon ps\_pos, pprarg\_neg targets |
|  | Model of PPARG signaling in psoriasis |
|  | PPARG negative regulators and targets |
|  | 3\_Enhanced angiogenesis in endometriosis |

---

|  |  |
| --- | --- |
| Group | Chemokines, Growth Factors and Receptors Involved in Cancer Cells Chemotaxis |

---

|  |  |
| --- | --- |
| MedScan ID | 2321 |

---

|  |  |
| --- | --- |
| LocusLink ID | 2321 |
|  | 14254 |
|  | 54251 |

---

|  |  |
| --- | --- |
| Alias | FMS-like tyrosine kinase I |
|  | Vascular endothelial growth factor receptor I |
|  | embryonic receptor kinase 2 |
|  | sFlt I |
|  | VEGF receptor 1 |
|  | Vascular endothelial growth factor receptor 1 |
|  | Flt I |
|  | OTTMUSP00000023727 |
|  | VEGFR I |
|  | VEGFA receptor 1 |
|  | Tyrosine- protein kinase FRT |
|  | Fms like tyrosine kinase receptor |
|  | Vascular permeability factor receptor |
|  | vascular endothelial growth factor/vascular permeability factor receptor |
|  | VEGF-R1 |
|  | Vascular permeability factor receptors |
|  | VEGF-A receptor 1 |
|  | fms-related tyrosine kinase 1 (vascular endothelial growth factor/vascular permeability factor receptor) |
|  | fms-related tyrosine kinase I |
|  | soluble VEGFR1 variant 2 |
|  | VEGFR1 |
|  | Flt 1 receptor |
|  | VEGF receptor I |
|  | Tyrosine-protein kinase receptor FLT |
|  | VEGF receptor FLT1 |
|  | AI323757 |
|  | soluble VEGF receptor 1-14 |
|  | soluble VEGFR1 variant 21 |
|  | receptor Flt1 |
|  | FMS-like tyrosine kinase 1 |
|  | vasculotropin receptor 1 |
|  | VGR1\_HUMAN |
|  | FRT |
|  | VEGF-A receptor FLT1 |
|  | sFlt1 |
|  | Flt1 |
|  | VEGFA receptor FLT1 |
|  | FLT |
|  | FLT-1 |
|  | VEGFR-1 |
|  | tyrosine-protein kinase FRT |
|  | vascular endothelial growth factor receptor-1 |
|  | fms-related tyrosine kinase 1 |

---

|  |  |
| --- | --- |
| EC Number | 2.7.10.1 |

---

|  |  |
| --- | --- |
| GO ID | 0005524 |
|  | 0036326 |
|  | 0036327 |
|  | 0019838 |
|  | 0036332 |
|  | 0004714 |
|  | 0038085 |
|  | 0005021 |
|  | 0048514 |
|  | 0030154 |
|  | 0016477 |
|  | 0035924 |
|  | 0048598 |
|  | 0002548 |
|  | 1905563 |
|  | 0018108 |
|  | 0070374 |
|  | 0043406 |
|  | 0043410 |
|  | 0045766 |
|  | 0030335 |
|  | 0008284 |
|  | 0043552 |
|  | 0014068 |
|  | 0010863 |
|  | 0030949 |
|  | 0046777 |
|  | 0002040 |
|  | 0007169 |
|  | 0048010 |
|  | 0036323 |
|  | 0038084 |
|  | 0015629 |
|  | 0005768 |
|  | 0005615 |
|  | 0005925 |
|  | 0005887 |
|  | 0005886 |
|  | 0043235 |
|  | 0016301 |
|  | 0000166 |
|  | 0004672 |
|  | 0004713 |
|  | 0016740 |
|  | 0001525 |
|  | 0001569 |
|  | 0006935 |
|  | 0007275 |
|  | 0016310 |
|  | 1901534 |
|  | 0048597 |
|  | 0006468 |
|  | 0001666 |
|  | 0016021 |
|  | 0016020 |
|  | 0005634 |
|  | 0000186 |
|  | 0030522 |
|  | 0048661 |
|  | 0006940 |
|  | 0016772 |
|  | 0005794 |
|  | 0005737 |
|  | 0005730 |
|  | 0004716 |
|  | 0048146 |
|  | 0023014 |

---

|  |  |
| --- | --- |
| KEGG ID | hsa:2321 |
|  | mmu:14254 |
|  | rno:54251 |

---

|  |  |
| --- | --- |
| Organism | Homo sapiens {Organism urn:agi-taxid:9606} |
|  | Mus musculus {Organism urn:agi-taxid:10090} |
|  | Homo sapiens |
|  | Mus musculus |
|  | Rattus norvegicus |

---

|  |  |
| --- | --- |
| Mouse chromosome position | 5 87.01 cM |

---

|  |  |
| --- | --- |
| OMIM ID | 165070 |

---

|  |  |
| --- | --- |
| Rat chromosome position | 12p11 |

---

|  |  |
| --- | --- |
| Hugo ID | 3763 |
|  | HGNC:3763 |

---

|  |  |
| --- | --- |
| Human chromosome position | 13q12.3 |
|  | 13q12 |

---

|  |  |
| --- | --- |
| Swiss-Prot Accession | P17948 |
|  | L7RSL3 |
|  | P17948.2 |
|  | A0A0R4J0A4 |
|  | P35969 |
|  | P35969.1 |
|  | A3E342 |
|  | A3E344 |
|  | A8KA71 |
|  | B0LPF1 |
|  | B2BF46 |
|  | B2BF47 |
|  | B2BF48 |
|  | B3FR89 |
|  | B5A923 |
|  | F5H5L6 |
|  | O60722 |
|  | P16057 |
|  | Q12954 |
|  | O55094 |
|  | Q61517 |
|  | P53767 |

---

|  |  |
| --- | --- |
| PIR ID | A49636 |
|  | S09982 |
|  | I78875 |
|  | S49010 |
|  | I60598 |

---

|  |  |
| --- | --- |
| GenBank ID | NC\_000013 |
|  | XM\_017020485 |
|  | XP\_016875974 |
|  | NM\_002019 |
|  | NP\_002010 |
|  | NM\_001160030 |
|  | NP\_001153502 |
|  | XM\_011535014 |
|  | XP\_011533316 |
|  | NM\_001159920 |
|  | NP\_001153392 |
|  | NM\_001160031 |
|  | NP\_001153503 |
|  | NG\_012003 |
|  | AL138712 |
|  | AL139005 |
|  | CH471075 |
|  | EAX08431 |
|  | EAX08432 |
|  | CQ871317 |
|  | CAH56859 |
|  | CS172284 |
|  | CAJ33638 |
|  | D00133 |
|  | BAA00080 |
|  | EU332841 |
|  | ABY87530 |
|  | HD077716 |
|  | CBW44131 |
|  | JX512442 |
|  | AGC09589 |
|  | KF002712 |
|  | AGL73885 |
|  | KF002713 |
|  | AGL73886 |
|  | KF206396 |
|  | AGS56393 |
|  | KF206397 |
|  | KF206398 |
|  | AGS56394 |
|  | KF206399 |
|  | KF680656 |
|  | AHC08482 |
|  | KF680657 |
|  | AHC08483 |
|  | KF680658 |
|  | AHC08484 |
|  | KF680659 |
|  | AHC08485 |
|  | KF680660 |
|  | AHC08486 |
|  | KF680661 |
|  | AHC08487 |
|  | KF680662 |
|  | AHC08488 |
|  | KF680663 |
|  | AHC08489 |
|  | KF854284 |
|  | AHO49116 |
|  | KF854285 |
|  | AHO49117 |
|  | KF897924 |
|  | AHO49114 |
|  | KF897925 |
|  | AHO49115 |
|  | AB209050 |
|  | BAD92287 |
|  | AF063657 |
|  | AAC16449 |
|  | AK092472 |
|  | AK292936 |
|  | BAF85625 |
|  | AK300392 |
|  | BAG62125 |
|  | AK309901 |
|  | BC029849 |
|  | BC039007 |
|  | AAH39007 |
|  | BE180461 |
|  | BU151621 |
|  | BX504049 |
|  | CA396081 |
|  | DA844548 |
|  | DA851664 |
|  | DQ836394 |
|  | ABI53803 |
|  | DQ836395 |
|  | ABI53804 |
|  | DQ836396 |
|  | ABI53805 |
|  | EF484674 |
|  | EF491868 |
|  | ABS32268 |
|  | EF491869 |
|  | ABS32269 |
|  | EF491870 |
|  | ABS32270 |
|  | EU360600 |
|  | ACB05747 |
|  | EU368830 |
|  | ACA62948 |
|  | EU443152 |
|  | ACA21474 |
|  | EU826561 |
|  | ACF47597 |
|  | EU826562 |
|  | ACF47598 |
|  | JF509744 |
|  | AFC77954 |
|  | JF509745 |
|  | AFC77955 |
|  | S77812 |
|  | AAB34001 |
|  | S77814 |
|  | AAB34002 |
|  | U01134 |
|  | AAC50060 |
|  | P17948 |
|  | NC\_000071 |
|  | NM\_010228 |
|  | NP\_034358 |
|  | XM\_011241000 |
|  | XP\_011239302 |
|  | XM\_006504801 |
|  | XP\_006504864 |
|  | XM\_006504802 |
|  | XP\_006504865 |
|  | XM\_006504803 |
|  | XP\_006504866 |
|  | NM\_001363135 |
|  | AC122299 |
|  | AC131730 |
|  | AF473823 |
|  | AAQ05293 |
|  | AJ224863 |
|  | CH466614 |
|  | EDL05837 |
|  | EDL05838 |
|  | EDL05839 |
|  | AJ001177 |
|  | CAA04571 |
|  | AK004721 |
|  | BAB23504 |
|  | AK005502 |
|  | BAB24084 |
|  | AK013236 |
|  | BAB28733 |
|  | AK035771 |
|  | AK084177 |
|  | AK146906 |
|  | BAE27519 |
|  | AK167497 |
|  | BAE39574 |
|  | AK167596 |
|  | BC029674 |
|  | AAH29674 |
|  | BP763953 |
|  | D88689 |
|  | BAA24498 |
|  | D88690 |
|  | BAA24499 |
|  | L07297 |
|  | AAA40078 |
|  | X78568 |
|  | CAA55311 |
|  | P35969 |
|  | NC\_018924 |
|  | AMYH02027768 |
|  | AMYH02027769 |
|  | AC\_000027 |
|  | AAHY01050787 |
|  | AAHY01050788 |
|  | AAHY01050789 |
|  | AAHY01050790 |
|  | AAHY01050791 |
|  | AAHY01050792 |
|  | AAHY01050793 |
|  | AAHY01050794 |
|  | AAHY01050795 |
|  | AAHY01050796 |
|  | AAHY01050797 |
|  | AAHY01050798 |
|  | AC\_000145 |
|  | ABBA01035724 |
|  | ABBA01035725 |
|  | ABBA01035726 |
|  | NC\_005111 |
|  | NM\_019306 |
|  | NP\_062179 |
|  | AC\_000080 |
|  | AABR06070259 |
|  | AABR06070260 |
|  | AAHX01071023 |
|  | AAHX01071024 |
|  | AAHX01071025 |
|  | AAHX01071026 |
|  | AAHX01071027 |
|  | CH474012 |
|  | EDL89555 |
|  | EDL89556 |
|  | AF157595 |
|  | AAF80356 |
|  | D28498 |
|  | BAA05857 |
|  | P53767 |

---

|  |  |
| --- | --- |
| Swiss-Prot ID | VGFR1\_HUMAN |
|  | VGFR1\_MOUSE |
|  | VGFR1\_RAT |

---

|  |  |
| --- | --- |
| Cell Localization | Cell membrane |
|  | Endosome |
|  | Secreted |
|  | Cytoplasm |
|  | Plasma membrane |

---

|  |  |
| --- | --- |
| Ensembl ID | ENSG00000102755 |
|  | ENSP00000484039.1 |
|  | ENST00000615840.4 |
|  | ENSP00000437631.1 |
|  | ENST00000541932.5 |
|  | ENSP00000442630.1 |
|  | ENST00000539099.1 |
|  | ENSP00000282397.4 |
|  | ENST00000282397.9 |
|  | ENSMUSG00000029648 |
|  | ENSMUSP00000031653.5 |
|  | ENSMUST00000031653.11 |
|  | ENSMUSP00000106158.1 |
|  | ENSMUST00000110529.5 |
|  | ENSP00000484039 |
|  | ENST00000615840 |
|  | ENSP00000437631 |
|  | ENST00000541932 |
|  | ENSP00000442630 |
|  | ENST00000539099 |
|  | ENSP00000282397 |
|  | ENST00000282397 |
|  | ENSMUSP00000031653 |
|  | ENSMUST00000031653 |
|  | ENSMUSP00000106158 |
|  | ENSMUST00000110529 |

---

|  |  |
| --- | --- |
| MGI ID | MGI:95558 |
|  | 95558 |

---

|  |  |
| --- | --- |
| RGD ID | 2621 |

---

|  |  |
| --- | --- |
| Unigene ID | Hs.594454 |
|  | Mm.389712 |
|  | Rn.10239 |

---

|  |  |
| --- | --- |
| Homologene ID | 134179 |
|  | 20463 |

---

|  |  |
| --- | --- |
| Shape | Stick-vertex |

---
